# The Immunomodulatory effect of exosomes in diabetes: a novel and attractive therapeutic tool in diabetes therapy

**DOI:** 10.3389/fimmu.2024.1357378

**Published:** 2024-04-24

**Authors:** Na Li, Lingli Hu, Jingyang Li, Yang Ye, Zhengyang Bao, Zhice Xu, Daozhen Chen, Jiaqi Tang, Ying Gu

**Affiliations:** ^1^ Research Institute for Reproductive Health and Genetic Diseases, Wuxi Maternity and Child Health Care Hospital, Wuxi, Jiangsu, China; ^2^ Graduate School of Nanjing Medical University, Nanjing, Jiangsu, China; ^3^ Institute for Fetology, First Affiliated Hospital of Soochow University, Suzhou, Jiangsu, China; ^4^ Department of Obstetrics, Wuxi Maternity and Child Health Care Hospital Affiliated to Nanjing Medical University, Wuxi, Jiangsu, China

**Keywords:** exosomes, diabetes, anti-inflammation, immune cells, clinical application

## Abstract

Exosomes carry proteins, metabolites, nucleic acids and lipids from their parent cell of origin. They are derived from cells through exocytosis, are ingested by target cells, and can transfer biological signals between local or distant cells. Therefore, exosomes are often modified in reaction to pathological processes, including infection, cancer, cardiovascular diseases and in response to metabolic perturbations such as obesity and diabetes, all of which involve a significant inflammatory aspect. Here, we discuss how immune cell-derived exosomes origin from neutrophils, T lymphocytes, macrophages impact on the immune reprogramming of diabetes and the associated complications. Besides, exosomes derived from stem cells and their immunomodulatory properties and anti-inflammation effect in diabetes are also reviewed. Moreover, As an important addition to previous reviews, we describes promising directions involving engineered exosomes as well as current challenges of clinical applications in diabetic therapy. Further research on exosomes will explore their potential in translational medicine and provide new avenues for the development of effective clinical diagnostics and therapeutic strategies for immunoregulation of diabetes.

## 1 Introduction

Diabetes mellitus, a group of metabolic disorders characterized by prolonged high blood sugar levels, is a global health issue affecting over 400 million people worldwide (1). This number is expected to surge to approximately 700 million by 2045 (2). The disease occurs either due to insufficient insulin production by the pancreas or the body’s inability to effectively utilize the produced insulin (3). The most common symptoms include weight loss, polydipsia, polyuria, and constant hunger. If not properly managed, diabetes mellitus can lead to severe complications such as kidney failure, unhealed wounds, vision loss, heart attacks, nerve damage, and even increase the risk of cancer (4). There are three main types of diabetes: type 1 diabetes mellitus (T1DM), type 2 diabetes mellitus (T2DM), and gestational diabetes mellitus. T1DM and T2DM account for 7-12% and 85-90% of global diabetes cases respectively. The rapid increase in diabetes mellitus cases worldwide underscores the disease’s significance as a public health concern.

Besides traditional treatment with insulin and oral anti-diabetic drugs, clinicians are attempting to enhance patient care through the use of cell therapies involving embryonic stem cells (ESC), induced pluripotent stem cells (iPSC), and adult mesenchymal stem cells (MSC) (5). However, there are unintended safety concerns such as immune rejection, genetic or disease transfer, and ectopic cell differentiation existing in whole-cell therapy. Recently, exosomes have been reported to play a role in multiple diseases and have been shown to be key mediators of various pathogenetic mechanisms. Compared with cell-based therapy, exosomes contain large amounts of bioactive molecules including proteins and nucleic acids. They exhibit high biocompatibility and low immunogenicity (6), and are able to circulate into distant sites and freely pass across the blood-brain barrier duo to their nanoscale size (7).

Recent studies have shown that exosomes play a role in the occurrence, development, and treatment of diabetes and its complications. However, there are few summaries from the perspective of immunity and inflammation regarding the treatment and mechanisms of exosomes from different cell sources in diabetes and its complications. This review summarizes the latest advances concerning the roles of exosomes and immune regulation/inflammation in diabetes.

## 2 Description of exosomes

Exosomes are small membrane-bound vesicles secreted by cells, usually between 30 and 200 nanometers in diameter. They play an important role in transmitting information between cells, regulating cell function, and participating in the occurrence and development of diseases (8). The biogenesis of exosomes involves three processes: generation, release, and uptake (9). Within the cells, membrane proteins and lipid molecules responsible for membrane synthesis are synthesized and packaged into endoplasmic reticulum vesicles. Subsequently, these vesicles fuse into polyvesicles. Vesicles in polyvesicles can further fuse to form exosomes (9). The release of exosomes is mainly accomplished through the fusion of polyvesicles with cell membranes. When the polyvesicles fuse with the cell membrane, the inner vesicles are released outside the cell to form exosomes (10). Exosomes are taken up by target cells by means of membrane fusion and endocytosis, and then release their cargo into the cytoplasm to exert their effects (11).Therefore, exosomes may manipulate recipient cells and other organs over a long distance (12).

Previous studies have demonstrated that exosomes, functioning as intercellular junctions, transport proteins, lipids, and nucleic acids to target cells. They are involved in a variety of biological processes including nucleic acid regulation, antigen presentation, metabolite transportation, and inflammation management. Furthermore, they hold potential as diagnostic and therapeutic tools for various diseases (13). Significantly, small non-coding RNAs (ncRNAs), which are approximately 19 to 24 nts in length and are a subset of nucleic acids, have garnered considerable interest within the scientific community due to their regulatory function (14). In this review, we have summarized the involvement of exosomes derived from immune cells and non-immune cells (such as stem cells) in the occurrence and intervention mechanisms of diabetes and its complications, many of which involve ncRNAs (**Table 1**), based on recent reports. Thus, delivery of multiple ncRNAs via exosomes may have promise over a wide range of applications.

**Table 1:** Changes of exosomal ncRNAs in diabetes.

Source	Models	Contents	Alteration	Functions	References
adipose tissue macrophages	T2DM	miR-210	increase	promoted diabetes pathogenesis by regulating glucose uptake and mitochondrial CIV activity	(15)
adipose tissue macrophage	T2DM	miR-29a	increase	induced insulin resistance	(16)
M1 macrophage	T2DM	miR-212-5p	increase	restricted insulin secretion	(17)
bone marrow-derived macrophages	T2DM	miR-144-5p	increase	impaired bone regeneration	(18)
macrophage	Diabetic vascular disease	miR-150-5p	decrease	promoted resistin expression in macrophages	(19)
M2 macrophages	Diabetic nephropathy	miR-93-5p	increase	attenuated LPS-induced podocyte apoptosis	(20)
EPCs	Diabetic wounds	miRNA-221-3p	increase	downregulated the expression of p27 and p57 proteins in the cell cycle signaling pathway	(21)
EPCs	Diabetic wounds	miR-126-3p	increase	promoted the recovery of tubulogenic function of high-glucose-impaired HUVECs.	(22)
EPCs	Diabetic stroke	miR-126	increase	attenuated acute injury and promoted neurological function recovery	(23)
EPCs	Diabetic wounds	mmu_circ_0000250	increase	enhanced the therapeutic effect of ADSC-exosomes to promote wound healing	(24)
ADSC	Diabetic wounds	miR-132	increase	reduced inflammation, promoting angiogenesis and stimulated M2-macrophages polarization, promote wound healing	(25)
ADSC	Diabetic wounds	miR-21-5p	increase	induced M2 polarization of macrophages and augmented skin wound healing	(26)
HypADSCs	Diabetic wounds	miR-21-3p/miR-126-5p/miR-31-5p	increase	promoted diabetic wounds healing and inhibited inflammation	(27)
HypADSCs	Diabetic wounds	miR-99b/miR-146-a	decrease	promoted diabetic wounds healing and inhibited inflammation	(27)
MSCs	Diabetic kidney disease	miR-424-5p	increase	alleviated high glucose-induced cell apoptosis and EMT	(28)
MSCs	Diabetic kidney disease	miR-22-3p	increase	protected podocytes and reduced inflammation	(29)
MSCs	Diabetic nephropathy	miR-146a-5p	decrease	restored renal function, facilitated M2 macrophage polarization	(30)
MSCs	Retinal inflammation	miR-126	decrease	reduced high glucose-induced HMGB1 expression and the activity of the NLRP3 inflammasome	(31)
MSCs	Diabetic wounds	miR -155	increase	NA	(32)
MSCs	Diabetic foot ulcer	lncRNA H19	decrease	prevented the apoptosis and inflammation of fibroblasts, leading to the stimulated wound-healing process	(33)
MSCs	Diabetic wound	lncRNA KLF3-AS1	increase	down-regulated miR-383, boosted expression of VEGFA	(34)
MSCs	Diabetic stroke	miR-9	decrease	promoted white matter remodeling and anti-inflammatory responses	(35)

EPCs, endothelial progenitor cells; ADSC, adipocyte-derived stem cell; HypADSCs, hypoxia adipose stem cell; MSCs, mesenchymal stem cells; T2DM, type 2 diabetes mellitus; CIV,continuous intravenous infusion; LPS, lipopolysaccharide; HUVECs, human umbilical vein endothelial cells; EMT, epithelial-mesenchymal transition; HMGB1,high mobility group box 1 protein; NLRP3, nod-like receptor thermal protein domain associated protein 3; VEGFA, vascular endothelial growth factor A; NA, not applicable.

## 3 Immune cell-derived exosomes and diabetes

In 1996, Raposo et al. reported that B lymphocytes secrete antigen-presenting vesicles (36). Since then, more and more studies have found that exosomes secreted by immune cells interact with cells in the immune system to regulate immune responses (37). Therefore, these membranous vesicles are being explored as potential immunotherapeutic reagents. Immune cell-derived exosomes can activate the immune system through various mechanisms (38). Firstly, they can directly activate immune cells such as dendritic cells and T cells through antigen presentation on their surface. Secondly, they can indirectly activate immune cells by releasing immune-stimulating molecules such as cytokines and chemical mediators. In addition, immunogenic exosomes may also regulate the function of immune cells by transferring immune-related nucleic acid molecules such as miRNA and mRNA. Previous studies have shown that immune-derived exosomes played a role in the development and progression of diabetes mellitus, making them a key regulator in the disease (39).

### 3.1 The roles of neutrophils-derived exosomes in diabetes

Polymorphonuclear neutrophils (PMNs), which make up 40-70% of all white blood cells in humans, are the most prevalent type of granulocytes. Neutrophils act as the first line of defense against invasive pathogens in the host and have a natural ability to phagocytose pathogens (40). Thus, neutrophils serve as important immune and secretory cells and play a crucial role in inflammation and infection processes (41). The status of the parent cell is reflected in the neutrophils-EXOs, which exhibit strong antibacterial ability due to the presence of components like myeloperoxidase, elastase, dermcidin, and lysozyme (42). In a recent research, investigators loaded extracellular matrix (ECM) hydrogel with vascular endothelial growth factor (VEGF)-encapsulated activated neutrophil exosome mimetics (aPMNEM) to develop VEGF-aPMNEM-ECM hybrid hydrogel for treating chronic diabetic wounds (40). Compared to directly using exosomes or using exosomes derived from other cells, this aPMNEM-ECM based biomaterial has the following advantages (1): for wound infection treatment, aPMNEM can play an antibacterial role via bactericidal-associated proteins (2); as a carrier, aPMNEM can deliver cytokines, and protect them from degradation (3); as a hermosensitive material, ECM can function as an *in situ* gel *in vivo* and increase the residence of aPMNEM. The study not only provided a functional biomaterial for the regeneration of chronic diabetic wounds but also created a promising platform for cytokine therapy, which can potentially be used to treat different diseases by loading various available cytokines in aPMNEM-ECM (40).

### 3.2 The roles of T lymphocytes-derived exosomes in diabetes

Type 1 diabetes mellitus is an autoimmune disorder characterized by infiltration of the islets of Langerhans by immune cells and by selective elimination of the insulin-secreting β cells (43). Regazzi’s team reported that miR-142-3p, miR-142-5p and miR-155 are particularly enriched in T lymphocytes of 8 weeks NOD mice with respect to mouse pancreatic islets (44). In type 1 diabetes, T lymphocytes-EXOs carrying specific microRNAs that induce chemokine expression and apoptosis in recipient pancreatic β cells. The inactivation of miR-142-3p/-5p and miR-155 in β cells leads to increased insulin levels, decreased insulitis scores, reduced inflammation, and provides protection against diabetes development in NOD mice (44).

### 3.3 The roles of macrophages-derived exosomes in diabetes

**Figure 1:**
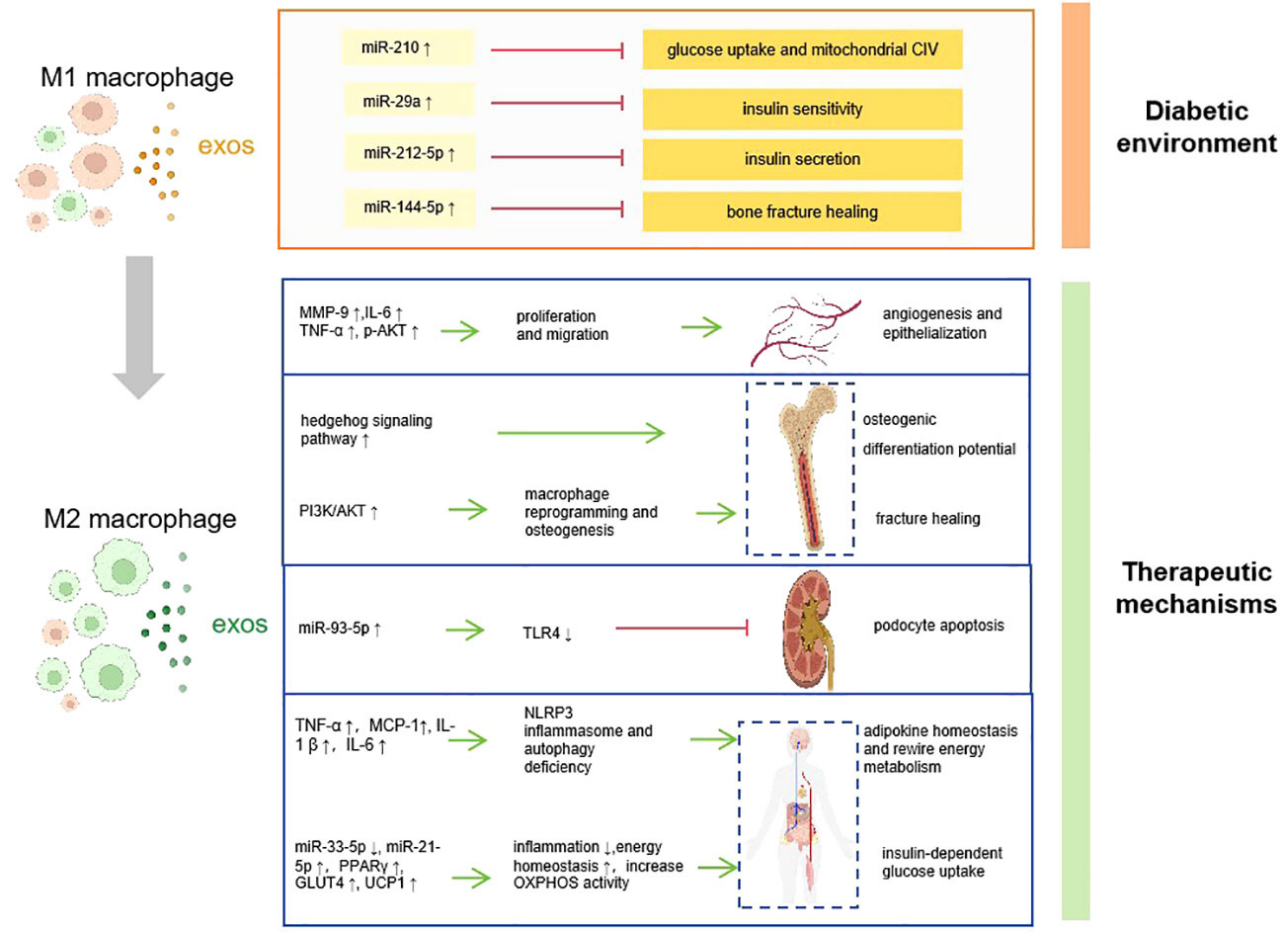
How macrophage derived-exosomes contribute to the pathogenesis, complications, and therapy of diabetes. Diabetic environment induce macrophage to M1 polarization, and the M1 macrophage secret exosomes which contains abnormal ncRNAs that promote diabetes and associated complications. Converting the ratio of M1/M2 macrophage polarization is supposed to be a therapeutic application, which accelerates diabetes recovery via various mechanisms. CIV, complex IV; MMP-9, matrix metalloproteinase-9; IL-6, interleukin-6; TNF-α, tumor necrosis factor-α; p-AKT, phospho-Akt; PI3K, phosphoinositide 3-kinase; TLR4, toll-like receptor 4; MCP-1, monocyte chemotactic protein-1; interleukin-1β; NLRP3, NOD-like receptor thermal protein domain associated protein 3; PPARγ, peroxisome proliferator-activated receptor γ; GLUT4, glucose transporter type 4; UCP1, uncoupling protein 1; OXPHOX, oxidative phosphorylation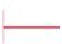:inhibit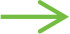:promote.

Macrophage-derived exosomes have been shown to have diverse functions in immune regulation, tissue repair, and communication between cells (45). Based on the functional profiles, macrophages are divided into two sub-populations: type 1 macrophages (M1, pro-inflammation) and type 2 macrophages (M2, anti-inflammation) (46). M1 macrophages play a role in the early phase of inflammation and are linked to tissue damage and pro-inflammatory activities, whereas M2 macrophages release cytokines that suppress inflammation and have anti-inflammatory effects (47). Recent studies have shown that the macrophages-EXOs contribute to the progression of diabetes (48) (**Figure 1**).

#### 3.3.1 Exosomes derived from M1 macrophages

##### 3.3.1.1 Impairing insulin sensitivity, secretion and glucose uptake through miRNAs

Chronic tissue inflammation caused by accumulation of M1 macrophages is an important hallmark of insulin resistance. According to prior research, the population of activated M1 macrophages residing within adipose tissue increased in obese mice, resulting in an increased ratio of M1 to M2 macrophages (49). The M1 macrophage is the predominant cell responsible for secreting exosomes containing miR-29a in obese mice (16). MiR-29a targets peroxisome proliferator-activated receptor-δ, leading to impairments of insulin sensitivity both *in vitro* and *in vivo* (16). Moreover, M1 macrophage secreted exosomal miRNA may directly give rise to beta cell impairment. Qian et al. reported that the M1 macrophage-EXOs contained miR-212-5p, which regulated the Protein Kinase B (Akt)/Glycogen synthase kinase3β (GSK-3β)/β-catenin pathway in receptor beta cells by targeting the sirtuin 2 gene to restrict insulin secretion (17). Thus, targeting miRNA or inhibiting M1 macrophage-EXOs could be manipulated to inhibit beta cell injury in T2DM.

##### 3.3.1.2 Promoting autophagy deficiency and resistin expression

It was found that high glucose stimulation promoted the polarization of macrophages to the M1-phenotype and produced more exosomes, thereby inducing activation of NOD-like receptor thermal protein domain associated protein 3 (NLRP3) inflammasome and autophagy defects in mesangial cells, promoting development of diabetic nephropathy (50). Besides, exosomal miR-7002-5p are highly expressed in high glucose treated macrophages, which suppress autophagy activity through targeting Atg9b in mouse tubular epithelial cell and C57 mouse kidney (51). In addition to regulate functions of kidney, macrophage-derived exosomes shows impact on diabetic vascular diseases. For example, under high glucose conditions, macrophage-derived exosomal metastasis associated lung adenocarcinoma transcript 1 (MALAT1) is upregulated, inhibiting the expression of miR-150-5p and counteracting its inhibitory effect on macrophage resistance factor expression, and promoting vascular diseases. Thus, macrophage-EXOs containing MALAT1 may serve as a novel target for diabetic vascular diseases (19).

##### 3.3.1.3 Impairing bone fracture healing

Patients with diabetes have an increased risk of nonunion and delayed union of fractures. Exosomes derived from diabetic bone marrow-derived macrophages (dBMDM-EXOs) transfer miR-144-5p to bone marrow stromal cells, inhibiting the expression of Smad1, thereby reducing bone repair and regeneration both *in vivo* and *in vitro* (18). Suppression of miR-144-5p remarkably reversed the adverse effects of dBMDM-EXOs on the osteogenic potential and the ability of fracture repair (18). However, the author didn’t test the ratio of M1/M2 or confirm the phenotype of the macrophages that transferred specific miRNAs. Given the function of M1 macrophages, they may be the predominant cell responsible for secreting exosomes containing miR-144-5p, which can lead to bone impairment.

#### 3.3.2 Exosomes derived from M2 macrophages (M2 macrophages-EXOs)

M2 macrophages release cytokines that play a role in anti-inflammatory and tissue repair (47). Previous data validate the association between treatment of diabetic-related diseases and the exosomes secreted by M2 macrophages. For example, the M2 macrophages-EXOs reduced lipopolysaccharides-induced podocyte apoptosis by regulating the miR-93-5p/TLR4 axis, which provided a new perspective for the treatment of diabetic nephropathy patients (20). Tuan et al. Demonstrated (52) that M2 macrophage-EXOs could control chronic inflammatory diseases caused by excessive energy storage. Interleukin 4 (IL-4) stimulated THP-1 macrophage-derived extracellular vesicles can improve the homeostasis of adipose factors, retargeting the energy metabolism of macrophages and adipocytes, thereby controlling the occurrence of cardiac metabolic tissue inflammation in obesity-related diabetes.

In addition to diabetic nephropathy and cardiac diseases, M2 macrophage-EXOs are necessary for accelerating diabetic bone fracture healing. A research has shown that M2 macrophage-EXOs can activate the Hedgehog signaling pathway in BMSCs in a high glucose and high insulin microenvironment, promoting osteogenic differentiation. This suggests that they can serve as a new approach for reshaping the immune homeostasis in diabetic bone (53). Additionally, the research has demonstrated that M2 macrophage-EXOs induced the transformation of M1 macrophages into M2 macrophages by stimulating the phosphoinositide 3-kinase (PI3K)/AKT pathway, significantly reducing the proportion of M1 macrophages and regulating the bone immune microenvironment, thereby accelerating diabetic bone fracture healing (54).

## 4 Exosomes derived from stem cell and their effect on immune/inflammation in diabetes

**Figure 2:**
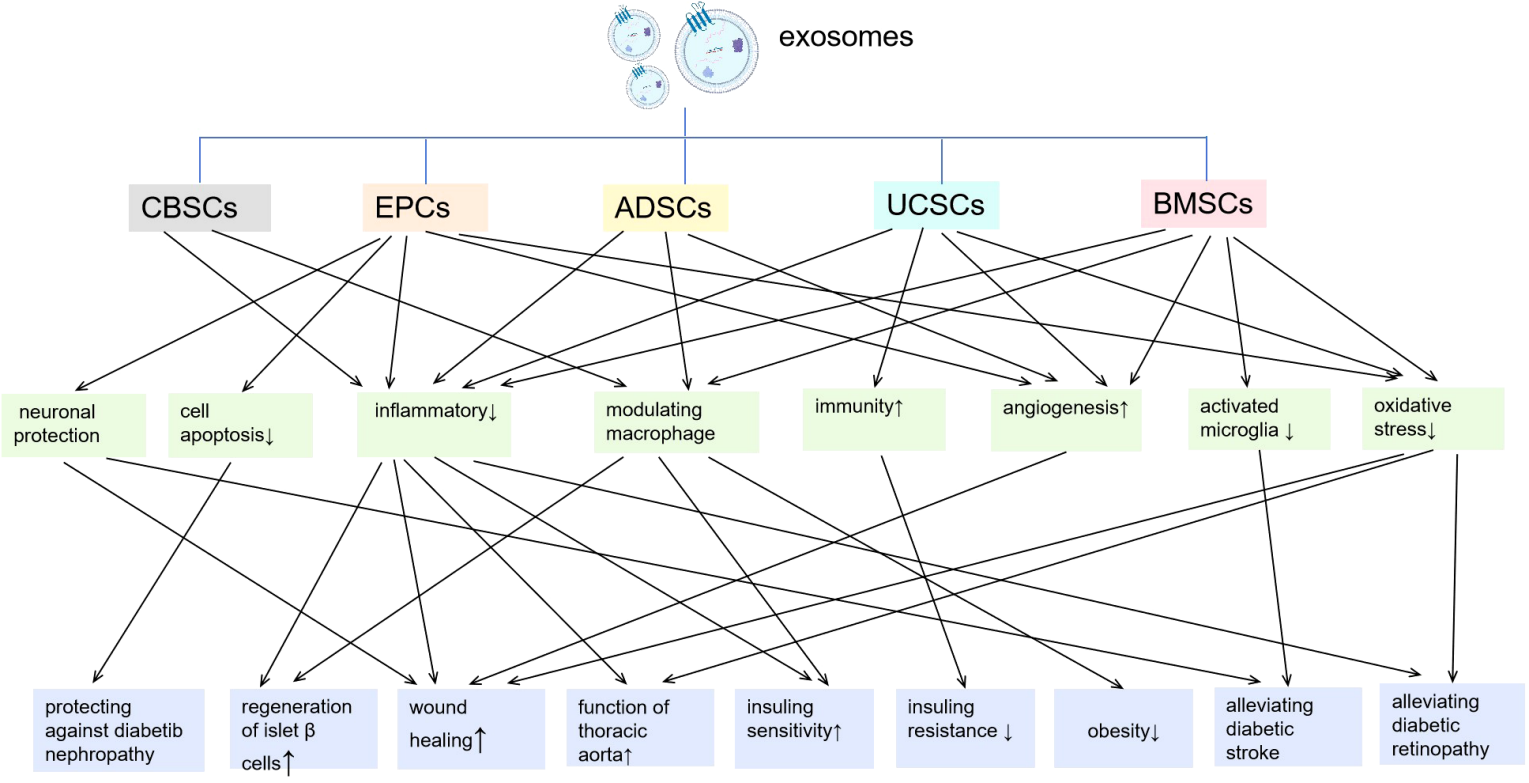
Exosomes from different kinds of stem cells and their main mechanisms underlying regulatory effects on inflammation/immunity in diabetes. CBSCs, cord-blood-derived stem cells; EPCs, endothelial progenitor cells; ADSCs, adipose stem cells; UCSCs, umbilical cord mesenchymal stem cells; BMSCs, bone marrow-derived mesenchymal stem cells.

In recent years, exosomes-based therapy have gained increasing attention for their comparatively high safety, biocompatibility and low immunogenicity (6). This part reviewed the exosomes from different kinds of stem cells and their main mechanisms underlying regulatory effects on inflammation/immunity in diabetes (**Figure 2**).

### 4.1 Cord-blood-derived stem cells

Cord blood-derived stem cells are multipotent stem cells that exhibit a distinct phenotype characterized by both embryonic and hematopoietic markers, distinguishing them from other known stem cell types (55, 56). Phenotypic characterization reveals that CBSCs exhibit embryonic cell markers. Moreover, CBSCs exhibit minimal immunogenicity, as evidenced by their low expression of major histocompatibility complex (MHC) antigens and their inability to stimulate the proliferation of allogeneic lymphocytes (55, 57). Specifically, CBSCs adhere firmly to culture dishes, displaying a large rounded morphology, and are resistant to common detachment methods (trypsin/EDTA), facilitating the collection of suspended lymphocytes after co-culture (55, 57). Based on the unique properties of immune modulation mentioned above and their ability to adhere tightly to the surface of Petri dishes, a new technology called Stem Cell Educator (SCE) therapy was designated for use in clinical trials (58, 59). Stem Cell Educator therapy (Educator therapy) has been utilized with a closed-loop system and open-loop system. During SCE therapy, a patient’s peripheral blood mononuclear cells (PBMCs) are collected and circulated through a cell separator, where they are co-cultured with adherent human CBSCs *in vitro*. The resulting “educated” cells, known as CBSC-treated PBMCs, are then reintroduced into the patient’s circulation (60). These “educated” immune cells can educate other immune cells after infusion, thereby reverse the root cause(s) of the autoimmune disease and resulting in the long-lasting clinical efficacy of Educator therapy. Unlike traditional immune therapies, SCE therapy does not destroy the cells responsible for autoimmunity but modifies them (61). The clinical phase 1/2 trials indicate that SCE therapy reverses autoimmunity, promotes regeneration of islet β cells, and improves metabolic control for the treatment of Type 1 diabetes (59, 62, 63) and T2DM (59, 63).

Mechanistic studies revealed that the secretion of CBSC-derived exosomes (CBSC-EXOs) enabled polarization of human blood monocytes/macrophages into M2 macrophages, thereby fundamentally correcting self-immunity and inducing immune tolerance through various molecular and cellular mechanisms (60). CBSC-EXOs preferably and quickly bind to monocytes within 2-3 h. During the coculture of CBSCs with patient’s immune cells for clinical treatment during 8-9 h, the SCE-treated monocytes may transport the CBSC-EXOs back into the body, potentially leading to additional M2 differentiation and induction of tolerance (59, 62). Therefore, Educator therapy is the leading immunotherapy to date to safely and efficiently correct autoimmunity through CBSCs mediated immune modulation and anti-inflammatory clinical effects, without the safety and ethical concerns associated with conventional immune and/or stem-cell based approaches.

### 4.2 Endothelial progenitor cells

Chronic diabetic foot ulceration (DFU) is among the most debilitating long-standing diabetes complications and it is also one of the main causes of physical disability. DFU is partially a result of unregulated foot wound infection caused by neuropathy, hindered angiogenesis, chronic low-grade inflammation, and peripheral vascular/arterial disease (64). Prolonged hyperglycemia intensifies the expression of inflammatory cytokines and reactive oxygen species (ROS), which severely impede angiogenesis (65–67). Thus, wound healing in diabetes always heavily relies on the formation of new blood vessels to restore reperfusion (68). EPCs are the precursors of endothelial cells, which hold great potential in treating chronic non-healing diabetic wounds because of their abilities for vascular and neuronal protection, repair and regenesis (69, 70). Nevertheless, the direct utilization of stem/progenitor cells is constrained by concerns such as potential immunological rejection, chromosomal variation, and emboli formation (71–73). Therefore, it is crucial to devise a new approach that can maximize the therapeutic benefits of stem/progenitor cells while mitigating the risks associated with their direct application.

It has been reported that the exosomes derived from EPCs (EPC-EXOs) can regulate vascular endothelial cells through miRNA. For example (21), EPC-EXOs exhibited a high expression of miRNA-221-3p. Treating skin wounds in diabetic mice with EPC-EXOs demonstrated a similar effect to that seen with miRNA-221-3p administration. MiRNA-221-3p potentially downregulated critical proteins in the AGERAGE signaling pathway, inhibiting reactive oxygen species generation and inactivating nuclear factor-kappa B (NF-kB). This process may reduce inflammatory responses, cell apoptosis, and microvascular diseases. Except for miRNA-221-3p, recent results revealed that treatment with miR-126-3 overexpressing EPC-EXOs accelerated the healing of rat skin wounds and resulted in better tissue repair with slower scar formation. In this process, the expression of caspase-1, NRLP3, interleukin-1b, inteleukin-18, PIK3R2 and SPRED1 was suppressed, promoting diabetic wound repair (22).

Exosomes derived from EPCs were reported to promote angiogenesis and the homing ability of EPCs in diabetic wound healing. Li et al. treated a diabetic rat wound model with EPC-EXOs and found that exosomes enhanced the proliferation, migration and tube formation of vascular endothelial cells *in vitro*. Furthermore, endothelial cells stimulated with EPC-EXOs showed increase expression of angiogenesis-related molecules such as fibroblast growth factor-1 (FGF-1), VEGFA, VEGFR-2, angiotensin I, E-selectin, Chemokine (C-X-C motif) ligand-16 (CXCR-16), endothelial nitric oxide synthase and IL-8 (74). In addition to promoting angiogenesis in wound healing, microvesicles derived from EPCs were demonstrated to be capable of changing the properties of adipose stem cells (ADSCs), thereby, improving their homing ability to migrate to the wound site. Tu TC et al. transfected exosomes derived from Alde-Low EPCs (EMVs) into human ADSCs. After receiving EMVs, the ADSCs showed a remarkable elevation in the expression of the CXCR4 chemokine receptor *in vitro*, and CD45+ inflammatory cells were successfully recruited to the wound sites *in vivo*, promoting ischemic skin repair (75).

Diabetes mellitus not only increases the risk of ischemia-reperfusion by 3-4 times compared to those without diabetes mellitus, but also exacerbates cerebral damage due to impaired endothelial function and reduced angiogenesis (23). EPCs were demonstrated to hold great potential in the treatment of stoke due to the cerebrovascular protection in the acute phase and promoting neurological recovery in chronic phases (76, 77). Previously experiment in mice indicated that enrichment of miR126 enhanced the therapeutic efficacy of EPC-EXOs on diabetic ischemic stroke by attenuating acute injury and promoting neurological function recovery (23).

Moreover, EPC-EXOs could potentially be a potential therapeutic application for treating Aherosclerosis (AS) resulting from diabetes. AS is a major macrovascular complication of diabetes mellitus characterized by inflammation and endothelial damage (78). The dysfunction of the endothelium is considered an early marker of AS. EPCs are derived from bone marrow and can differentiate into endothelium cells. In cases where ECs are damaged, EPCs may replace them to assist in the recovery from endothelial dysfunction (79). It was demonstrated that EPCs-EXOs had a significant impact on reducing D-AS plaques, lowering the levels of inflammatory factors such as intercellular cell adhesion molecule-1, IL-8, and C-reactive protein, decreasing oxidative stress factors like malondialdehyde and superoxide dismutase, and improving the function of thoracic aorta vasodilation and constriction in a mouse model of diabetic AS (80).

### 4.3 Mesenchymal stem cell

Mesenchymal stem cells possess various biological characteristics, such as immunomodulation, anti-inflammatory properties, and promotion of angiogenesis, making them widely used in clinical treatment and regenerative medicine (81). MSC-EXOs have been shown to be similar effective as MSCs in the treatment of diabetes and related complications (82–84), but in some contexts, they exert different biological properties (85).

#### 4.3.1 Adipose stem cells

Adipocyte-derived stem cells have been attracting attention as an effective therapeutic tool for tissue regeneration. Exosomes derived from ADSCs (ADSC-EXOs) can ameliorate inflammation by regulating immune cells, thereby promoting the treatment of diabetes and its related complications.

##### 4.3.1.1 ADSC-EXOs modulate macrophage polarization and immune cell activities in diabetes

Zhao et al. demonstrated that treatment with ADSC-EXOs improved metabolic homeostasis in obese mice, including enhanced insulin sensitivity (27.8% improvement), reduced obesity, and alleviated hepatic steatosis. ADSC-EXOs induced M2 macrophage polarization, reduced inflammation, and promoted Beiging in white adipose tissues (WAT) of diet-induced obese mice. Such exosomes carried active signal transducer and activator of transcription 3 (STAT3), which facilitated arginase-1 expression in macrophages, leading to the induction of anti-inflammatory M2 phenotypes. Additionally, the M2 macrophages induced by ADSC-EXOs stimulated ADSC proliferation and lactate production, thereby promoting WAT beiging and maintaining homeostasis in response to high-fat challenge (86). Luo et al. reported that overexpression of hematopoietic prostaglandin D synthase HPGDS in ADSCs accelerated chronic wound healing by improving the anti-inflammatory state and promoting M2 macrophage polarization in type 2 diabetic mice (87). As for M1 macrophages, ADSCs-EXOs play an immunosuppressive role by reducing IFN-α secretion, thus inhibiting activation of T cells, leading to enhanced aggregation capacity of M1 macrophages (88, 89). Besides, ADSC-EXOs promoted T-regulatory cell activation and facilitated wound healing by inhibiting interferon-g production and M1 macrophage accumulation in an EFGR signal-dependent manner (90).

Moreover, recent research found ADSC-EXOs to be a vital source of non-coding RNA to enhance M2 macrophage polarization and promote diabetic wound healing. For example, hypoxic treatment significantly increased circ-Snhg11 contents in ADSC-EXOs and promoted M2 polarization by inhibiting miR-144-3p expression and the STAT3 signaling pathway in skin wounds (91, 92). In another study, the *in vivo* experiment demonstrated that exosomes derived from miR-132-overexpressing ADSC significantly improved the survival of skin flaps and accelerated diabetic wound healing. This was achieved by reducing local inflammation, promoting angiogenesis, and stimulating M2 macrophage polarization through the NF-κB signaling pathway (25). Li et al. found that treating diabetic foot ulcer wounds with ADSC-EXOs increased miR-21-5p levels in macrophages, promoted M2 polarization, and inhibited Keuppel-like factor 6 KLF6, which has been reported to enhance the inflammatory phenotype in macrophages (26).

These findings delineate novel exosome-mediated mechanisms for ADSC-macrophage crosstalk that facilitates immune and metabolic homeostasis, thus providing potential therapy for obesity and diabetes.

##### 4.3.1.2 ADSC-EXOs revers the inflammatory condition in wound healing

Wound healing can be delayed by chronic and excessive inflammation, therefore a well-regulated inflammation guarantees wound healing (88). ADSCs-EXOs contain immunoregulatory proteins such as tumor necrosis factor-α (TNF-α), macrophage colony-stimulating factor and retinol-binding protein 4 (93). In addition to the local effects, ADSC-EXOs can reverse the systematic inflammatory condition in diabetes models. Qiu et al. demonstrated that high glucose treatment significantly increased inflammatory factors IL-6, IL-1β, and TNF-α levels in EPCs from healthy volunteers. Such elevated levels could be partially and completely reversed by ADSC-EXOs and linc00511-overexpressing ADSCs (94). They found Exosomes from linc00511-overexpressing ADSCs promotes diabetic foot ulcers healing by accelerating angiogenesis via suppressing PAQR3-induced Twist1 ubiquitin degradation as well as suppressed inflammatory. Zhang et al. found that ADSC-EXOs significantly reduced levels of inflammatory cytokines IL-6, TNF-a, and monocyte chemotactic protein-1 (MCP-1) by decreasing ROS production and protecting mitochondrial function via sirtuin-3 (95). Wang et al. found that hypoxic ADSC-EXOs exhibited distinct miRNA expression profiles compared to ADSC-EXOs. Specifically, up-regulation of miR-21-3p, miR-126-5p, and miR-31-5p, and down-regulation of miR-99b and miR-146-a in hypoxic ADSC-EXOs promoted wound healing in diabetic mice and suppressed inflammatory factors through the PI3K/AKT signaling pathway (27). Shi reported that exosomes derived from mmu_circ_0000250-modified ADSCs promoted wound healing in diabetic mice by inducing miR-128-3p/SIRT1-mediated autophagy and improving the hyperglycemic-induced inflammatory microenvironment and recover the function of EPCs (24).

#### 4.3.2 Umbilical cord mesenchymal stem cells

Human umbilical cord tissue (Wharton’s jelly) serves as a potent and rich source of MSCs. UCSCs-derived exosomes (UCSC-EXOs) have shown promising results in the treatment of diabetes and may become a successful strategy for treating diabetes and its complications. Injection of UCSC-EXOs significantly ameliorated hyperglycemia in rats with T2DM (96). Besides, UCSC-EXOs also contributes to the therapy of other diabetic complications, such as diabetic nephropathy, retinopathy and wound ulcer.

##### 4.3.2.1 UCSC-EXOs increase insulin sensitivity by suppress inflammatory factors

Chronic inflammation in tissues is typically the primary cause of insulin resistance, which results in the secretion of pro-inflammatory cytokines such as tumor necrosis factor alpha (TNF-α) or IL-6 by inflammatory cells. These cytokines then inhibit the activation of the insulin signaling pathway (97, 98). It is found that injection of human UCSC-EXOs significantly ameliorated hyperglycemia in rats with T2DM. UCSC-EXOs could increase insulin sensitivity by increasing the activation of insulin/AKT signaling pathway and inhibiting the secretion of proinflammatory cytokines like TNF-α, which could reverse insulin resistance in T2DM (96).

##### 4.3.2.2 The role of UCSC-EXOs in diabetic nephropathy

It is demonstrated that UCSC-EXOs could be a promising treatment strategy for diabetic nephropathy rats. Xiang et al. reported that UCSC-EXOs apparently reduced the levels of pro-inflammatory cytokines (IL-6, IL-1β, and TNF-α) and pro-fibrotic factor transforming growth factor β (TGF-β) in the kidney and blood of diabetic nephropathy rats. *In vitro* experiments showed that umbilical cord MSC conditioned medium and UCSC-EXOs decreased the production of these cytokines in high glucose injured renal tubular epithelial cells, and renal glomerular endothelial cells (99). Besides, UCSC-EXOs miR-424-5p can inhibit the activation of yes associated protein 1 in HK2 cells, reduce cell apoptosis, and epithelial-to-mesenchymal transition induced by high glucose, thereby attenuating diabetic nephropathy (28). MiR-22-3p, highly expressed in UCSC-EXOs, may play a protective role in podocytes and diabetic mice by regulating the NLRP3 inflammasome. This suggests that MSC-derived exosomes could be a promising cell-free therapeutic strategy for diabetic kidney disease (29). Another study showed that UCSC-EXOs miR-146a-5p enhanced M2 macrophage polarization by inhibiting the TRAF6/STAT1 signaling pathway, thereby protecting against diabetic nephropathy in rats (30).

##### 4.3.2.3 The role of UCSC-EXOs in wound healing and diabetic retinopathy

UCSC-EXOs serve as a novel therapeutic approach to enhance wound healing in diabetes. Studies have shown that UCSC-EXOs can induce anti-inflammatory macrophages (100), leading to a reduction in the expression of inflammatory factors such as IL-1β, IL-6, and TNF-α (101), as well as promoting angiogenesis and collagen deposition. Furthermore, UCSC-EXOs have the potential to inhibit oxidative stress injury, thereby facilitating macro-level angiogenesis and ultimately expediting the healing of diabetic wounds (101).

In addition to diabetic wounds, diabetic retinopathy is another common complication of diabetes. Previous studies have shown the therapeutic effect of UCSC-EXOs in diabetic retinopathy. For example, the administration of miR-126-expressing UCSC-EXOs significantly reduced high glucose-induced high-mobility group box 1 expression and the activity of the NLRP3 inflammasome in human retinal endothelial cells, therefore suppressing suppressed inflammation in diabetic rats (31).

At last, UCSC-EXOs treatment could be beneficial for diabetic rats to recover from the anemia-like symptoms and increase immunity by improving the erythrocytes and hemoglobin levels as well as maintaining the number of white blood cells (102). 1 mg/kg of UCSC-EXOs improved glucose tolerance in T2DM rats and ameliorate insulin resistance. Moreover, there was no significant difference in white blood cells, neutrophils, lymphocytes, monocytes, eosinophils, and basophils between the diabetic rat groups treated with both glibenclamide (one of the traditional hypoglycemic drug) and 1 mg/kg of UCSC-EXOs and the non-diabetic animal group. This finding suggests that the administration of UCSC-EXOs at 1 mg/kg could improve the immune system of diabetic rats, which is essential for reducing infections and increasing survival rates (102).

#### 4.3.3 Bone marrow-derived mesenchymal stem cells

Bone marrow mesenchymal stem cells are multilineage progenitors with self-renewal, multidirectional differentiation, and pleiotropic paracrine functions (103). It is demonstrated that purified BMSC-derived exosomes (BMSC-EXOs) have more specific distinct benefits in damaged tissue repair than BMSCs themselves, including superior stability, tissue permeability, excellent biocompatibility, and immunomodulatory properties (104).

##### 4.3.3.1 The role of BMSC-EXOs in diabetic wound healing

Accumulative studies have shown that BMSC-EXOs contribute to wound healing through non-coding RNAs. For example, Liu et al. found that miR-155-inhibitor-loaded BMSC-EXOs enhanced keratinocytes migration, FGF-7 recovery, and anti-inflammatory effects *in vitro*. Additionally, they could also be utilized to treat a diabetic wound model by promoting collagen deposition, angiogenesis, and re-epithelization. The functional coordination between miR-155-inhibitor and BMSC-EXOs played a crucial role in enhancing diabetic wound healing (32). Li reported that the injection of BMSC-EXOs overexpressing lncRNA H19 facilitated wound healing in mice with diabetic foot ulcers. Results revealed that BMSC-EXOs overexpressing lncRNA H19 led to higher level of IL-10 and lower levels of IL-1b and TNF-a, and the mechanism by which was associated with promoting fibroblast proliferation and migration, inhibiting cell apoptosis and inflammation (33). In a murine diabetic cutaneous wound model, exosomes from lncRNA KLF3-AS1-expressing BMSCs demonstrated the best effects in promoting cutaneous wound healing in diabetic mice, which were associated with minimal weight loss, increased blood vessel formation, reduced inflammation, decreased miR-383 expression, and up-regulated VEGFA (34). Except for non-coding RNAs, the anti-inflammation effect by BMSC-EXOs could induced by specific pathways that may not directly related to non-coding RNAs. Wang reported that the wounds treated with exosomes showed reduced inflammation, with decreased levels of the inflammatory cytokines TNF-α and IL-1β, and increased levels of the anti-inflammatory cytokines IL-4 and IL-10 (105). Such regenerative and anti-inflammatory effects were eliminated by Lenti-sh-Nrf2 administration, suggesting the participation of the activation of Nrf2 anti-oxidant pathway in wound healing by exosomes. In addition to miRNAs, Liu et al. reported that melatonin-pretreated BMSC-EXOs could promote diabetic wound healing by suppressing the inflammatory response, which was achieved by increasing the ratio of M2 polarization to M1 polarization through activating the phosphatase and tensin homolog/AKT signaling pathway (106).

##### 4.3.3.2 The role of BMSC-EXOs in diabetic stroke

Diabetes increases the risk of stroke by 3-4 fold, and about 30% of stroke patients suffer from diabetes (107). Treating patients with diabetic stroke is challenging because it may cause extensive damage to the cerebral vasculature, exacerbate neurological deficits, enhance inflammatory responses, which are prone to recurrent strokes (108, 109). Therefore, it is crucial to devise therapeutic strategies specifically aimed at enhancing neurological function after stroke in individuals with diabetes. MSCs interact with and alter brain parenchymal cells via the secretion of trophic and growth factors as well as exosomes to exert therapeutic effects (110). Exosome therapy offers several advantages compared to cell therapy, as exosomes do not elicit immune rejection, do not cause vascular obstruction, and have a low risk of triggering tumors or malignant transformation (111). Besides, exosomes are more suitable for clinical use since they are relatively stable, can be obtained in large quantities from a small number of cells, and can be stored until therapeutic needed (112). Therefore, systemic administration of exosomes could be a method of delivering the active components of cell therapy to the central nervous system (113).

Studies (35, 114) have indicated that T2DM stroke was associated with increased inflammatory responses and proinflammatory microglial/macrophage phenotype. The inflammatory factor matrix metalloproteinase-9 (MMP-9) was elevated after stroke and has been implicated in aggravating blood-brain barrier disruption, neuronal death, myelin degradation and white matter injury. In addition, the inflammatory factor MCP-1 was elevated in the serum of both diabetic and stroke patients, and it aids in the accumulation of phagocytic M1 macrophages in the infarct border (115, 116). However, T2DM-BMSC-EXOs treatment has been demonstrated to significantly decrease activated microglia, M1 macrophage, and inflammatory factors MMP-9 and MCP-1 expression in the ischemic brain in T2DM stroke rats (35). Such therapeutic effects in neurological functional recovery were only induced by injection of exosomes derived from BMSCs of T2DM rats but not from BMSCs of non-diabetic animals, which may be partially mediated by decreasing miR-9 and upregulating ABCA1-IGFR1 pathway (35).

##### 4.3.3.3 The role of BMSC-EXOs in diabetic retinopathy

BMSCs-Exos also possess other immunomodulatory properties and can suppress the activation and function of various immune cells involved in islet transplantation and diabetic retinopathy. It is reported that co-delivery of siFas and anti-miR-375 by BMSCs and derived exosomes suppressed early apoptosis of transplanted human islets, while further immune activity could be suppressed by intravenously injection of human BMSC and PBMC co-cultured exosomes. Thus, BMSC and peripheral blood mononuclear cell co-cultured exosomes performed a immunosuppressive effect for improving islet transplantation (117). Besides, BMSC-EXOs improve diabetes-induced retinal damage by inhibiting the Wnt/β-catenin signaling pathway, subsequently reducing oxidative stress, inflammation, and angiogenesis (118). BMSC-EXOs miR-146a regulates the inflammatory response of diabetic retinopathy by mediating the TLR4/MyD88/NF-κB pathway, reducing the levels of TNF-α, IL-1β, and IL-6 (119).

## 5 Exosomes as an innovative therapeutic tools for diabetes: current status and promising directions

### 5.1 Promising directions

Exosomes exhibit high biocompatibility and low immunogenicity, which makes them have great potential in delivering nucleic acid sequences and chemotherapy drugs (6). However, studies have shown that the natural half-life of most exosomes *in vivo* is relatively short (<6 h) (120), and the contents of natural exosomes are limited by the secreting cells, resulting in limited therapeutic effects when loaded with drug molecules. To date, increasing researches demonstrated that under certain stress or modified conditions, stem cells can produce more exosomes or exosomes with different compositions compared to basal conditions. Meanwhile, many studies demonstrated the beneficial effects of modified or pretreated stem cell-derived exosomes on preventing comorbidities or microvascular complications in diabetes. These benefits mainly stem from the following three perspectives (**Table 2**): a. Exosomes from genetically modified stem cells display enhanced effects on diabetic wound healing compared to wild-type exosomes; b. By adding specific drugs to the culture medium, cells may secrete exosomes that are more effective in targeting angiogenesis, anti-inflammation, promoting proliferation and migration, and inhibiting apoptosis; c. Under certain stress conditions, such as hypoxia, cells may secrete exosomes that perform better in promoting fibroblast proliferation and migration, and enhancing reepithelialization in chronic wounds. All the above demonstrated that preconditioning or pre-treatment of diabetic MSCs with various agents/stress can be used to optimize/improve cellular function prior to their use in cell therapy.

**Table 2:** Pre-intervention to improve the function of exosomes in the treatment of diabetes.

Disease and animal	Cell type releasing Exo	Intervention	Pathways	Effect: *in virto*	Effect: *in vivo*	Effect on inflammation /immune system	ref
Diabetic cutaneous wound, Rat	hAMSCs	miR-21-5p overexpressing	Wnt/β-catenin pathways ↑	proliferation and migration of keratinocyte cells ↑	vessel growth and maturing ↑, wound healing process ↑	inflammatory cell infiltration↓	(121)
Diabetic wound, Mice	hAMSCs	hypoxia	PI3K/Akt pathways ↑	fibroblast proliferation and migration ↑	re-epithelialization ↑	CD31↑, TGF-β ↑, COLI ↑ and COLIII ↑, IL-6 ↓	(27)
Diabetic full-thickness excisional wound, Mice	ADSCs	mmu_circ_0000250-overexpressing	miR-128-3p/SIRT1 pathway↑	HG-induced EPC apoptosis ↓, autophagy of EPC ↑	wound closure ↑	SIRT1-mediated anti-inflammatory ↑	(24)
Diabetic foot ulcer, Mice	ADSCs	mmu_circ_0001052 overexpressing	miR-106a-5p ↓, FGF4/p38MAPK pathway ↑	proliferation ↑, migration and angiogenesis of high glucose-induced HUVEC ↑	speed of healing ↑	NA	(122)
Diabetic foot ulcer, Rat	ADSC	Nrf2 overexpression	SMP30 ↑, VEGF ↑, p-VEGFR2 ↑, ROS ↓	increased cell viability ↑, tube formation of EPCs ↑	Ulcerated area ↓, angiogenesis ↑, inflammation ↓, oxidative stress ↓	IL-1β ↓, IL-6 ↓, TNF-α ↓	(123)
Diabetic full-thickness wounds, Rat	BMSC	atorvastatin pretreated	AKT/eNOS pathway ↑	endothelial cell angiogenesis↑	Ascularization ↑ , the wound healing ↑	NA	(124)
Diabetic full thickness dermal dorsal defect, Rat	BMSC	pioglitazone-pretreated	PI3K/AKT/eNOS pathway ↑	migration and tube formation ↑, wound repair ↑, VEGF expression of HUVEC ↑	diabetic wound healing ↑, angiogenesis ↑	NA	(125)
Diabetic full-thickness dermal defect, Rat	BMSC	melatonin-pretreated	PTEN/AKT pathway ↑	ratio of M2 polarization to M1 polarization in RAW264.7 cells ↑	angiogenesis and collagen synthesis ↑	ratio of M2 / M1 polarization ↑,IL-1β ↓, TNF-α ↓, IL-10 ↑, Arg-1 ↑	(106)
Diabetic punch biopsy excisional wound, Mice	BMSC	HOTAIR overexpressing	NA	HOTAIR ↑,VEGF ↑ in endothelial cells	angiogenesis ↑ and wound healin ↑	NA	(125)
Diabetic foot ulcer, mice	BMSC	lncRNA H19 overexpression	miR-152-3p-mediated PTEN inhibition ↓	apoptosis and inflammation of fibroblasts ↓	flammatory cells ↓, granulation tissues thicker around the wound	IL-10 ↑, IL-1b ↓, TNF-a ↓	(33)
diabetic wounds rat	HEK293	miR-31-5p overexpression	HIF1AN ↓, EMP-1↓	cell proliferation ↑ and migration ↑ in ECs, HFF-1 cells and HaCaT cells; capillary-like construction activity ↑ in ECs	proangiogenesis ↑, profi ↑, brogenesis ↑, reepithelization↑	NA	(126)
Diabetic cutaneous wound, Rat	UC-MSC	Lipopolysaccharide-pretreated	M2 macrophage polarization ↑ through let-7b via TLR4/NF-κB/STAT3/AKT pathway	converted inflammatory THP-1 cells to M2 polarization	inflammatory cell infiltration ↓, new small capillaries and woundhealing ↑	anti-inflammatory cytokines ↑, M2 macrophage activation ↑	(127)

hAMSCs, human adipose-derived mesenchymal stem cells; ADSCs, adipocyte-derived stem cells; ADSC, adipocyte-derived stem cell; BMSC, bone mesenchymal stem cells; HEK293, human embryonic kidney 293T cells; UC-MSC, Umbilical cord-derived mesenchymal stem cells; PI3K, phosphatidyl-inositol 3-kinase; AKT, protein kinase b; SIRT1, silent information regulator 1; FGF4, fibroblast growth factor 4; p38MAPK, P38 mitogen-activated protein kinase; SMP30, senescence marker protein 30; VEGF, vascular endothelial growth factor; VEGFR2 , vascular endothelial growth factor receptor 2; ROS, reactive oxygen species; eNOS, endothelial nitric oxide synthase; NA, ot applicabl; HIF1AN, hypoxia inducible factor 1 subunit alpha inhibitor; EMP-1, EPO mimetic peptide-1; TLR4, toll-like receptor 4; NF-κB,nuclear factor kappa-B; STAT3, Signal transducer and activator of transcription 3; EPC, endothelial progenitor cells; HUVEC, human umbilical vein endothelial cells; VEGF, vascular endothelial growth factor; HOTAIR, HOX transcript antisense RNA; ECs, early career specialists; THP, human monocytic-leukemia cells; CD31, platelet endothelial cell adhesion molecule-1; TGF-β, transforming growth factor β; COLI, Collagen I; IL-6, Interleukin 6; IL-1β, Interleukin-1β; TNF-α,Tumor Necrosis Factor-α; IL-10, Interleukin-10; Arg-1, Arginase 1; IL-1b, Interleukin-1β.

In addition to modify the donor cells that produce exosomes, direct modification to purified natural exosomes may efficiently and quickly obtain a large number of engineered exosomes, and reduce the uncertainty in the cell culture process, which is of great significance for the mass production of engineered exosomes. For example, taking advantage of natural availability and biocompatibility of exosomes as extracellular miRNA transporting particles (121), Lv et al. reported a human hASC-exos-based miRNA delivery strategy which loaded miRNA into hASC-exos by electroporation. Besides electroporation, other physical methods such as ultrasonic homogenization (128), freeze-thaw cycle (129), may also allow drugs to enter the exosomes more easily, achieving the purpose of engineering exosomes. However, such methods were usually used in treatment of cancers *in vitro* or *in vivo* in animal models, therefore, future research will focus more on the application of these methods in the treatment of diabetes and the associated complications.

Finally, in recent years, due to the high biocompatibility and modifiability, composite hydrogels loaded with exosomes and other nanoparticles have gained increasing attention in managing chronic diabetic wounds. Compared to traditional stem cell therapy, which has been shown to have short survival times, poor stability, and a high risk of immune rejection in diabetic ulcers (130), exosomes-loaded composite hydrogels have been demonstrated to possess superior functions in angiogenesis, anti-inflammatory, antibacterial, and antioxidant properties (**Table 3**). Since different agents have varying applicability, advantages and disadvantages for wound healing, various therapeutic agents can be incorporated inside the multifunctional hydrogel to create an outstanding drug delivery system (143). Thus, the exosomes-loaded, “all-in-one” composite hydrogels may achieve a controlled drug delivery in diabetic wound healing, prone to better drug applications.

**Table 3:** Functions of composite hydrogels in the treatment of diabetic wound healing (2020 to date).

Publication year	Cell type releasing EXOs	Hydrogels	Anti-inflammatory effect	Antibacterial effect	Angiogenesis	Antioxidant effect	ref
2020	CBSCs	PF-127 hydrogel	inflammatory cell infiltration ↓	unknown	TGFβ-1 ↑, VEGF ↑	unknown	(130)
2022	M2Φ	HA-based hydrogels composed of MnO2 and FGF-2	unknow	+	angiogenic ability ↑	ameliorated ROS damage	(127)
2022	ADSCs	ADSC-exo@MMP-PEG smart	unknow	unknown	CD31 and α-SMA ↑, re-epithelialization and collagen deposition ↑	ROS level ↓	(131)
2022	HUVECs	GelMA/PEGDA@T+exos MNs patch	unknow	unknown	angiogenesis ↑	unknown	(132)
2022	BMSCs	carboxyethyl chitosan -dialdehyde carboxymethyl cellulose hydrogel	skewing macrophage M1 to M2 phenotype	+	Angiogenesis ↑, VEGF-mediated signaling pathways ↑	unknown	(133)
2022	ESCs	Gel-VH-EVs	unknow	unknown	angiogenesis ↑, HIF-1α-mediated pathway ↑	unknown	(134)
2023	ADSCs	hydrogel loaded with 4-Arm-PEG-Thiol, Ag^+^, exosomes, CNTs, and metformin hydrochloride	IL-6 ↓, TNF-α ↓, ICAM and VCAM ↓	+	density and quantity of blood vessels ↑	ROS and mtROS production ↓	(135)
2023	M2Φ	hydrogel combined with bioactive M2-Exos and gold nanorods	proinflammatory cytokines ↓	+	CD31+ ↑, vascular network formation ↑	SOD1 ↑, PRDX2 ↑	(136)
2023	ADSCs	extracellular matrix hydrogel	TNF-α ↓, IL-6 ↓	unknow	collagen deposition ↑, skin regeneration ↑, blood vessel numbers ↑	unknown	(136)
2023	PMN	VEGF-aPMNEM-ECM hybrid hydrogel	M1 macrophage transform to M2 macrophage ↑	+	number of blood vessels↑	unknown	(40)
2023	ADSCs	GelMA-Exo hydrogels	unknow	unknow	proliferation, invasion, and tube formation ↑	unknown	(137)
2023	HUVECs	ADM Fe3+@PA-Exos/GelMA	IL-1β ↓	+	proliferation and migration impairment ↓	SOD and GSH-Px activity ↑	(138)
2023	HUVECs	hypoxic exosomes-loaded HGM-QCS hydrogels	IL-6 ↓, TNF-α ↓,ICAM-1↓, SELE ↓, VCAM-1 ↓, M2 polarization ↑	+	collagen deposition ↑, angiogenesis ↑	ROS level ↓	(139)
2024	Umbilical cord blood	UCB-Exos into an ABA-type amphiphilic hydrogel	unknow	unknow	proliferation and tube formation ↑	unknown	(140)
2024	Whole blood	P-Exos-loaded CMC hydrogeL	unknow	unknow	angiogenesis ↑, VEGF mediated signaling pathways ↑	unknown	(141)
2024	hUC-MSCs	hydrogel composed of chitosan nanoparticles, MSC- derived, BG, and TiO2	TGF-β and IL-10 ↑, TNF-α ↓, IL-1β ↓, IL-6 ↓	+	enhanced angiogenesis of ECs by targeting VEGFA and VEGFR2	unknown	(142)

M2Φ, M2 macrophages; ADSCs, adipose-derived stem cells; HUVECs, human umbilical vein endothelial cells; BMSCs, bone marrow mesenchymal stromal cells; ESCs, embryonic stem cell; PMN, polymorphonuclear neutrophils; hUC-MSCs, human umbilical cord mesenchymal stem cells; MnO2, manganese dioxide; FGF-2, fibroblast growth factor-2; MMP, matrix metalloproteinases; PEG, polyethylene glycol; GelMA, gelatin methacryloyl; PEGDA, poly (ethylene glycol) diacrylate; IL-6, interleukin-6; TNF-α, tumor necrosis factor-α; ICAM, intercellular cell adhesion molecule; VCAM, vascular cell adhesion molecule; IL-1β, interleukin—1β; ICAM-1, intercellular cell adhesion molecule-1; VCAM-1, vascular cell adhesion molecule-1; TGF-β, transforming growth factor-β; IL-10, interleukin-10; VEGF, vascular endothelial growth factor; CD31, platelet endothelial cell adhesion molecule-1; α-SMA, α-smooth muscle actin; VEGFA, vascular endothelial growth factor A; VEGFR2, vascular endothelial growth factor receptor 2; ROS, reactive oxygen species; mtROS, mitochondrial reactive oxygen species; SOD1, recombinant superoxide dismutase 1; PRDX2, peroxiredoxin-2; GSH-Px, glutathione peroxidase; SOD, recombinant superoxide dismutase.

### 5.2 Current challenges of clinical applications

So far, there are mainly three challenges in the clinical translations of exosomes. Firstly, minimize the therapeutic efficacy differences caused by physiological and structural variations between human and animals. Exosomes derived from various stem cell sources have been used in wound healing treatments across animal models including mice (144, 145), rats (123), rabbits (146), consistently demonstrating positive effects such as improved wound closure, reduced healing time, enhanced angiogenesis, and diminished scar formation. However, the outcomes of these preclinical studies do not necessarily translate to human skin due to significant differences in skin structure and physiology, with pig skin being the closest analogue to human skin. Porcine models have emerged as promising models to study wound healing, they possess similar anatomically and physiologically characteristics to humans, including a relatively thick epidermis, distinct rete pegs, dermal papillae, and dense elastic fibers in the dermis (147), porcine collagen (148) et al. In contrast to rodent, rabbit, and canine skin, which exhibits loos adherence to the subcutaneous fascia, porcine skin closely adheres to the underlying structures, resembling human skin (149). The turnover time of pig epidermis is similar to the human epidermis (150). Moreover, the immune cells in pig skin resemble those found in human skin (151). According to research by Sullivan and colleagues, pig models were 78% concordant with human studies. This result exceeded other small-mammal and *in vitro* models, which were only 53% and 57% concordant (152). Therefore, it is crucial to validate the biological effects of exosomes on wound healing using a pig model.

Secondly, the clinical translation of engineered extracellular vesicles is urgently needed. So far, clinical applications of these exosomes are limited to only a few clinical trials exploring the therapeutic effects of stem cell-derived exosomes for diabetes and its complications, such as wound healing. According to data from ClinicalTrials.gov, to date, three completed clinical trials have utilized exosomes derived from plasma (NCT02565264), adipose tissue (NCT05475418), and mesenchymal stem cells (NCT05813379) for wound healing. Another (NCT04134676) has explored the use of stem cell-conditioned medium for chronic ulcer wounds. Apart from wound treatment, very few clinical trials have investigated the use of exosomes for other diabetic conditions [only one for Type 1 diabetes (NCT02138331)].

Thirdly, The scaling-up manufacture of “Good Manufacturing Practice” (GMP)-grade exosomes is the most difficult component in the clinical use of exosomes. Challenges in the further clinical application of exosomes include quality control, such as the cell-culture system, purification, characterization/physicochemical and biological properties of exosomes, as well as the establishment of a “gold standard” for potency assay. Thus, advances in scaling-up technology for GMP-compliant exosomes manufacturing will enhance the clinical applications of these entities for diabetes and the related complications in the near future.

## 6 Concluding remarks and future perspectives

As a promising candidate for novel cell free therapy, exosomes may be widely used as an alternative to stem cells in management of a variety of immunity-related diseases or inflammation response for maintenance of the microenvironment for tissue homeostasis and tissue regeneration upon injury. In this review article, we describe how immune cell-derived exosomes origin from neutrophils, T lymphocytes and macrophages impact on diabetes and the associated complications. We also discuss the stem cell-derived exosomes and their role in immunomodulatory and inflammation in the progress of diabetic complications. In addition, promising directions involving engineered exosomes as well as current challenges of clinical applications are reviewed. The enhanced properties of engineered exosomes have been verified in lab, which proves that they have great clinical application prospects. However, there is still a long way to go before commercial exosome products are ready for the market, due to the lack of clinical trials and quality control for scaling-up manufacture.

In addition to the above challenges, some questions remain unanswered, which needs more attention to be paid to in the future. For example, how do exosomes transferred specific miRNAs target the genes in recipient cells? Besides, studies about gestational diabetes mellitus (GDM) are still limited. Although researchers have found that some exosomal non-coding RNAs in peripheral blood may be early diagnostic markers for GDM, it is unknown how exosomes interact with the immune system and contribute to the pathophysiology of GDM. Nevertheless, we remain confident that the hurdles facing these innovative approaches will be surmounted and that they will do influence the treatment of diabetes.

## Author contributions

NL: Conceptualization, Funding acquisition, Writing – original draft, Writing – review & editing. LH: Data curation, Resources, Writing – original draft. JL: Data curation, Investigation, Project administration, Writing – original draft. YY: Data curation, Formal analysis, Methodology, Writing – original draft. ZB: Writing – review & editing. ZX: Conceptualization, Supervision, Writing – review & editing. DC: Supervision, Writing – review & editing. JT: Conceptualization, Funding acquisition, Investigation, Validation, Writing – review & editing. YG: Funding acquisition, Project administration, Resources, Writing – review & editing.
